# Metabolic Alterations in Preneoplastic Development Revealed by Untargeted Metabolomic Analysis

**DOI:** 10.3389/fcell.2021.684036

**Published:** 2021-08-03

**Authors:** Henna Myllymäki, Jeanette Astorga Johansson, Estefania Grados Porro, Abigail Elliot, Tessa Moses, Yi Feng

**Affiliations:** ^1^Centre for Inflammation Research, Queen’s Medical Research Institute, Institute for Regeneration and Repair, The University of Edinburgh, Edinburgh, United Kingdom; ^2^EdinOmics, SynthSys - Centre for Synthetic and Systems Biology, School of Biological Sciences, The University of Edinburgh, Edinburgh, United Kingdom; ^3^Edinburgh Cancer Research UK Centre, Institute of Genetics and Molecular Medicine, University of Edinburgh, Edinburgh, United Kingdom

**Keywords:** zebrafish, metabolome, HRAS, cancer metabolism, preneoplastic, untargeted metabolomics

## Abstract

Metabolic rewiring is a critical hallmark of tumorigenesis and is essential for the development of cancer. Although many key features of metabolic alteration that are crucial for tumor cell survival, proliferation and progression have been identified, these are obtained from studies with established tumors and cancer cell lines. However, information on the essential metabolic changes that occur during pre-neoplastic cell (PNC) development that enables its progression to full blown tumor is still lacking. Here, we present an untargeted metabolomics analysis of human oncogene HRAS^G12V^ induced PNC development, using a transgenic inducible zebrafish larval skin development model. By comparison with normal sibling controls, we identified six metabolic pathways that are significantly altered during PNC development in the skin. Amongst these altered pathways are pyrimidine, purine and amino acid metabolism that are common to the cancer metabolic changes that support rapid cell proliferation and growth. Our data also suggest alterations in post transcriptional modification of RNAs that might play a role in PNC development. Our study provides a proof of principle work flow for identifying metabolic alterations during PNC development driven by an oncogenic mutation. In the future, this approach could be combined with transcriptomic or proteomic approaches to establish the detailed interaction between signaling networks and cellular metabolic pathways that occur at the onset of tumor progression.

## Introduction

Altered metabolism is a hallmark of cancer development that underpins the uncontrolled growth and proliferation of malignant cells (Hsu and Sabatini, 2008; Hanahan and Weinberg, 2011; Pavlova and Thompson, 2016). The original observation over ninety years ago by Otto Warburg has guided the cancer metabolism research field to elucidate mechanisms that contribute to enhanced aerobic glycolysis in cancer cells (Warburg et al., 1927; DeBerardinis et al., 2007; Heiden Vander et al., 2011). Initially it was assumed that mitochondrial damage and hence reduced oxidative phosphorylation lead to increased glycolysis (Warburg, 1956), but now it is widely accepted that this might not be the case. Instead, cancer cells actively alter their metabolic pathways so as to adapt to an anabolic life style associated with growth and proliferation (Cantor and Sabatini, 2012; Commisso et al., 2013; Boroughs and Deberardinis, 2015; Davidson et al., 2016; Biancur et al., 2017). Oncogene activation often leads to constitutively active growth factor signaling, which directly reprograms cellular metabolism to favor the macromolecular synthesis necessary for supplying nucleotides, proteins, and lipids for biomass expansion and cell division (Ying et al., 2012; Son et al., 2013; Pavlova and Thompson, 2016). Metabolic adaptation driven by different oncogenes leads to specific nutrient requirements in different types of cancer cells (Yuneva et al., 2012; Mayers et al., 2016; Ding et al., 2021). Such nutrient “addiction” could be exploited for targeted therapy (White, 2013; Alkan et al., 2018; Jin et al., 2019; Najumudeen et al., 2021).

Although it is generally accepted that metabolic adaptation is a hallmark of cancer, very little is known as to whether this metabolic reprogramming occurs at the pre-neoplastic stage of tumor development *in vivo*. PNCs acquire some features of tumor cells such as oncogenic mutation and clonal expansion. It is reasonable to assume that since the PNC re-enters the cell cycle and proliferates in contrast to its quiescent normal counterpart, there might be a measurable change in its metabolism. A better understanding of the metabolic requirement for PNC expansion might therefore provide an avenue for designing tumor prevention strategies and identify metabolic markers for studying tumorigenesis.

Untargeted or global metabolomics approaches analyze diverse classes of small molecules of low molecular weight (hereafter metabolites) within a single sample using separation technologies, state-of-the-art instrumentation and advanced data processing workflows. With an ultimate aim of capturing as many metabolites as possible, untargeted metabolomics allows viewing of the global metabolome and reviewing both known and unknown metabolic changes within any biological system using relative quantification across sample groups (Schrimpe-Rutledge et al., 2016). Such acquisition of data without pre-existing knowledge is a major advantage for new hypotheses generation; however, owing to the diverse composition of the metabolome, sample preparation, separation method and instrumentation platform directly impact the results obtained. Liquid chromatography (LC) coupled to mass spectrometry (MS) is a leading technology for the analyses of a broad variety of both polar and non-polar metabolites, particularly in complex biological samples such as those derived from mammalian systems. The versatility in metabolite coverage and sensitivity of instrumentation allows the detection and semi-quantification of several hundred metabolites within a sample. By incorporating ion mobility spectrometry (IMS) in LC-MS-based untargeted metabolomics, structural features of metabolites in complex matrices can be obtained by accessing drift time (tD) and collision cross section (CCS) values (with IMS) in addition to retention time (tR, with LC) and mass-to-charge values (*m/z*, with MS). When combined, the four parameters (tR, *m/z*, tD, CCS) provide increased resolution and more accurate identification of metabolites in complex biological samples (Odenkirk and Baker, 2020). Untargeted metabolomics using LC-MS-IMS thus allows an unbiased comprehensive study of the metabolites within PNCs, and offers the potential to reveal metabolic adaptation in cancer biology beyond the pre-conceived ideas.

In this study, we used an inducible zebrafish larval skin pre-neoplastic development model driven by the prototype human oncogene HRAS^G12V^ and developed a protocol to extract metabolites from larval skin tissue; we then performed untargeted metabolomics using LC-MS-IMS workflows to identify differentially presented metabolites in pre-neoplastic larval skin. Our analysis of these very early stage preneoplastic tissues identified metabolic alterations in pyrimidine, purine and amino acid metabolism, which are changes often reported in cancers. This work establishes a proof of principle for using the zebrafish larval skin model to assess metabolic re-programming induced by oncogene activation in PNCs during tumor initiation.

## Materials and Methods

### Chemicals

Ammonia solution 0.88 SG, formic acid, Optima^TM^ LC/MS grade water, and Optima^TM^ LC/MS grade acetonitrile were purchased from Fisher Scientific (Loughborough, United Kingdom). Ammonium acetate was from Sigma-Aldrich (Gillingham, United Kingdom), and ammonium formate from Thermo Fisher Acros Organics (Geel, Belgium). (Z)-4-Hydroxytamoxifen (4-OHT) and MS222 (ethyl 3-aminobenzoate methanesulfonate salt, also called tricaine) were purchased from Sigma Aldrich (Gillingham, United Kingdom).

### Animal Husbandry

Adult zebrafish were maintained in the Bioresearch and Veterinary Services (BVS) Aquatic facility in the Queen’s Medical Research Institute, the University of Edinburgh. Housing conditions were similar to those described in the Zebrafish Book (Westerfield, 2000) with 14/10 h light/dark cycle and water temperature of 28.5°C. The water quality, hardness and conductivity were controlled daily, and the pH was maintained at 7–7.5. All experiments were conducted with local ethical approval from the University of Edinburgh and in accordance with United Kingdom Home Office regulations (Guidance on the Operations of Animals, Scientific Procedures Act, 1986).

### Zebrafish Lines

The zebrafish lines used in this study were generated in house by the Feng lab using Tol2 mediated transgenesis. Tg(krtt1c19e::KalTA4-ER^*T2*^; cmlc2::eGFP)^ed201^ (hereafter K19 Gal4 driver) drives basal keratinocytes (Lee et al., 2014) expression of the KalTA4-ER^T2^ transcription factor, together with cmlc2::eGFP for identification of transgene-containing larvae by GFP fluorescence in the myocardium (green heart). Tg(UAS::eGFP-HRAS^G12V^; cmlc2::eGFP)^ed203^ (hereafter UAS:RAS) carries an UAS driven eGFP-HRAS^G12V^ expressing cassette and a cmlc2 promoter driven eGFP expression cassette as selection marker. Tg(UAS::eGFP-CAAX; cmlc2::eGFP)^ed204^ (hereafter UAS:CAAX) carries an UAS driven eGFP-CAAX expressing cassette and a cmlc2 promoter driven eGFP expression cassette as selection marker.

### Zebrafish Breeding and Embryo Collection

Adult K19 Gal4 driver fish were set for pair mating with either UAS:RAS or UAS:CAAX fish in breeding tanks that contained a divider. The next morning, the dividers were removed, and the fertilized embryos were collected within the next 2 h to ensure synchronous development. The embryos were maintained in 90 mm petri dishes (maximum of 50 embryos/dish) containing 0.3 × Danieau’s solution (17.40 mM NaCl, 0.21 mM KCl, 0.12 mM MgSO_4_7H_2_O, 0.18 mM Ca(NO_3_)_2_, 1.5 mM HEPES), in a 28.5 °C incubator.

### Tamoxifen (4-OHT) Induction and Larval Selection

The detailed induction protocol is described in Ramezani et al. (2015). In brief, the induction solution consisted of 0.3 × Danieau’s solution with 5 μM 4-OHT and 0.5% DMSO to enhance penetration of 4-OHT. A 10 mM stock of 4-OHT dissolved in 96% ethanol was stored at −20°C protected from light. At 52 h post-fertilization (hpf), larvae were transferred to petri dishes containing 20 mL 4-OHT induction solution, at 50 larvae per dish. The larvae were then maintained at 28.5°C in the dark.

**FIGURE 1:**
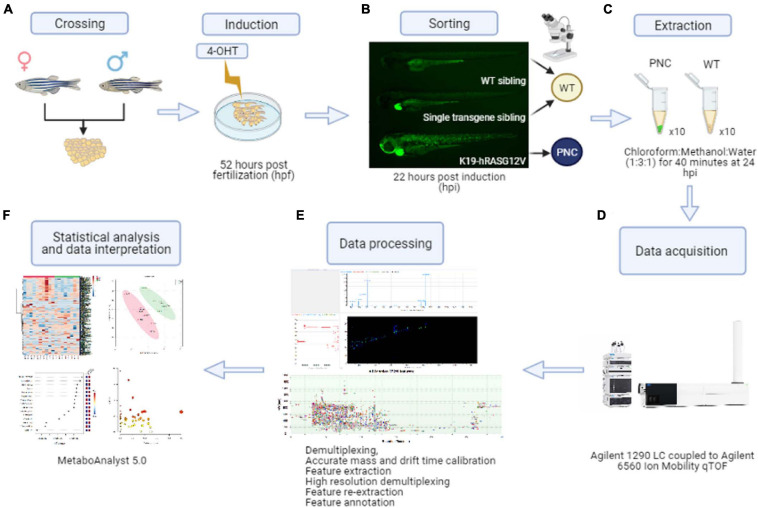
Preneoplastic cell (PNC) induction and schematic workflow for untargeted metabolomics from zebrafish larval skin. **(A)** Adult K19:Gal4 driver fish were crossed with UAS:RAS. Embryos were transferred to petri dishes containing 4-OHT induction solution at 52 hpf. **(B)** At 22 hpi larvae with eGFP positive skin cells were collected as the preneoplastic cell (PNC) group. Single transgene siblings (with a green heart but with GFP negative skin cells) and wild type siblings (negative for both green heart and skin cells) were collected as wild type sibling (WT) group. **(C)** Metabolite extractions were carried out at 24 hpi. **(D)** Metabolites were detected by liquid chromatography coupled to ion mobility mass spectrometry LC-MS-IMS instrumentation. **(E)** Data processing was performed using the Agilent MassHunter 10 software suite. Feature extraction was performed using Mass Profiler 10.0 on the demultiplexed raw data fist, and after High Resolution Demultiplexing prior to feature annotation. **(F)** Finally, statistical analysis and data interpretation was performed using the web-based software MetaboAnalyst 5.0.

At 22 h post-induction (hpi; corresponding to 74 hpf), the larvae were anaesthetized with MS222 and appropriate phenotypes were screened and sorted. Larvae with 30–60% coverage of GFP positive skin cells were collected as pre-neoplastic cell (PNC) group, and larvae with GFP negative skin were collected as Wild Type (WT) siblings group (Figure 1). During screening, the embryos were placed in fresh 4-OHT induction solution and the sorted larvae were maintained at 28.5°C in the dark for an additional period to yield a total induction time of 24 h.

### Metabolite Extraction From Zebrafish Larval Skin

For metabolite extraction, 10 biological replicates of the PNC group and sibling controls were used. The embryos were anaesthetized, washed twice with 0.3 × Danieau’s solution and transferred into 1.5 mL eppendorf tubes at 25 embryos/tube. Any remaining liquid was carefully removed by pipetting and 150 μL of ice-cold extraction buffer (chloroform:methanol:ddH_2_O, 1:3:1; chilled at −20°C prior to use) was added. The samples were placed on ice during handling and incubated at 4°C, 80 rpm for 40 min for extraction with further mixing after 15 and 30 min of incubation. The metabolite extracts had a yellowish color at the end of incubation while larvae remained intact. Samples were vortexed briefly, at high speed and centrifuged (15,000 × g) for 10 min at 4°C. The supernatants were transferred to pre-cooled 1.5 mL eppendorf tubes, which were immediately placed on dry ice. The samples were stored at −80°C until transported for LC-MS-IMS analysis on dry ice.

### EdU Labeling and Whole-Mount Immunostaining

EdU labeling was carried out using the Click-iT Plus EdU Alexa Fluor 647 Imaging Kit (Life Technologies, C10640, Thermo Fisher Scientific, Loughborough, United Kingdom) as described previously (van den Berg et al., 2019). Briefly, at 62 hpf (10 hpi), 74 hpf (22 hpi), or 86 hpf (34 hpi), 1 nL of 10 mM EdU (5-ethynyl-2′-deoxyuridine, a nucleoside analog of thymidine) was injected into the yolk of the larvae. After 2.5 h incubation at 28.5°C, the larvae were fixed in 4% paraformaldehyde (PFA) for 30 min at room temperature (12 and 24 hpi samples) or o/n at 4°C (36 hpi samples). The larvae were permeabilized in phosphate buffered saline (PBS) containing 0.5% Triton X-100 (PBST) for 5 min three times, and blocked with PBST containing 3% (w/v) Bovine Serum Albumin (Sigma-Aldrich, Gillingham, United Kingdom) for 1 h. This was followed by 30 min incubation with the Click-it Plus reaction cocktail according to manufacturer’s instructions, using 250 μL of reaction cocktail per max 10 larvae. Following EdU labeling, larvae were washed with PBST for 5 min three times and re-blocked with PBST containing 5% (v/v) Goat Serum (Sigma-Aldrich, Gillingham, United Kingdom) for 2 h. eGFP immunostaining was performed with rabbit monoclonal anti-GFP antibody (1:200; cat. 2956, Cell Signaling Technology, London, United Kingdom) and Alexa Fluor 488 Goat anti-Rabbit secondary antibody (1:250; A-11008, Invitrogen, Thermo Fisher Scientific, Loughborough, United Kingdom) as described (van den Berg et al., 2019). Stained larvae were stored at 4°C in a glycerol based antifadent mountant (AF1, CitiFluor, Hatfield, PA, United States) until mounted for imaging.

### Confocal Imaging, Data Analysis, and Statistical Analyses

Confocal imaging was performed using a Leica TCS SP8 AOBS confocal laser scanning microscope attached to a Leica DMi8 inverted microscope, with a 20 × dry lens, and excitation lasers 405, 488, and 633 nm were used. Percentage of EdU positive cells within eGFP positive cells were manually counted using the Imaris 9.0 software. In addition, PNCs were assessed for elongated shape and the presence of filopodia-like structures. Statistical significance was determined by multiple *t*-test using the Holm-Sidak method (EdU staining) and one-way ANOVA with Tukey’s multiple comparisons test (PNC morphology) using Prism 6 (GraphPad). Z projections of representative image stacks were generated using Fiji software (Schindelin et al., 2012).

### Gene Expression Analysis of Flow Cytometry Sorted PNC and CAAX Cells

4-OH tamoxifen induced zebrafish larvae were screened for fluorescently labeled HRAS and CAAX and anesthetised with MS-222. The two groups of larvae, HRAS and CAAX control were dissociated using collagenase IV (25 mg/mL) in medium containing goat serum (2% v/v), HBSS, HEPES (15 mM) and D-glucose (25 mM). The two samples were incubated for 3 × 5 min at 28.5°C until fully dissociated. The cell suspensions were diluted with an equal volume of medium containing goat serum (10% v/v) in HBSS, HEPES, D-glutamine and kept cold on ice. DAPI was added to distinguish dead cells and the samples were sorted on a BD FACS Aria II instrument using a 100 μm nozzle, selecting for fluorescently labeled HRAS or CAAX populations. Batches of 1,000 cells were collected in Eppendorf tubes containing 4 μL 0.2% Triton X and RNAase inhibitor (Thermo Fisher Scientific). The samples were processed for cDNA synthesis using a modified Smart-seq2 protocol (Picelli et al., 2014). Post clean-up, the cDNA quality was assessed using a LabChip GX instrument. Gene expression was analyzed using LightCycler 480 SYBR Green I Master (Roche) on a LightCycler 96 system using the following primers; sat1a.2_for 5′-CC GTTTTATCACTGCGTCGT-3′ and sat1a.2_rev 5′-AAAGC AGCTTCCCAATCCAG-3′, *itpa* for 5′-AGCCTGAGGGCTAT GACAAA-3′ and *itpa*_rev 5′-GTTTTGAGCGCTTGGTCT CT-3′, pnp5b_for 5′-TTCTCAGTCTTCATGCGGGA-3′ and *pnp5b*_rev 5′-CACCTCATGAACAGTGCTCA-3′, rrm2_for 5′-ACTGGGCACTCAACTGGATT-3′ and *rrm2*_rev 5′-GTC CCCTCTTCTTTAGCCAGA-3′.

### Chromatography and Ion Mobility Mass Spectrometry Method

Chromatographic separation was performed using a SeQuant ZIC-pHILIC, 5 μm polymeric 4.6 mm × 150 mm column (Merck KGaA 1.50461.0001, Darmstadt, Germany) coupled to a SeQuant ZIC-pHILIC Guard 20 × 2.1 mm column (Merck KGaA 1.50437.0001, Darmstadt, Germany). Two different solvent systems of low and high pH were used to run a 30-min gradient. The solvent system for acquiring data in the positive ionization mode consisted of 10 mM ammonium formate in water with 0.1% formic acid, pH 3 (solvent A) and 10 mM ammonium formate in water/acetonitrile (1:9) with 0.1% formic acid, pH 3 (solvent B). Similarly, the solvent system for acquiring data in negative ionization mode consisted of 10 mM ammonium acetate in water, pH 9 (solvent A) and 10 mM ammonium acetate in water/acetonitrile (1:9), pH 9 (solvent B). The solvent gradient for both ionization modes consisted of 80% solvent B at the start of the run, which was reduced to 20% solvent B in 15 min, and further to 5% solvent B in 1 min, where it was maintained for 4 min. At 21 min the column was returned to initial conditions of 80% solvent B and maintained as such until 30 min. A constant flow rate of 0.3 mL/min was observed during the run and the column was maintained at 30°C. For analysis, 5 μL sample was injected into the column. A quality control sample generated by pooling equal volumes of each sample was injected five times at the beginning of the experiment to condition the column and after every five samples to monitor the instrument state over the course of data acquisition.

The LC-MS-IMS instrumentation consisted of an Agilent 1290 Infinity II series UHPLC system coupled to an Agilent 6560 IM-qTOF (both Agilent Technologies, Santa Clara, CA) with a Dual Agilent Jet Stream Electron Ionization source. In both ionization modes data was acquired in the 50-1700 *m/z* range, with an MS acquisition rate of 0.8 frames/s. The nebulizer pressure was set to 60 psi, gas temperature to 225°C and drying gas (N_2_) flow rate to 13 L/min. Sheath gas was set to 340°C with a flow rate of 12 L/min, and the instrument was operated at a capillary voltage of 3,000 V, nozzle voltage of 200 V, fragmentor voltage of 395 V, and octupole voltage of 750 V. Ionization mode parameters for the IMS are instrument specific and available upon request from the authors. Instrument calibration and tuning was performed separately for each ionization polarity using the ESI-L low concentration tuning mix from Agilent Technologies (Santa Clara, CA). A reference mass solution consisting of 50 μM ammonium trifluoroacetate, 5 μM purine and 1.125 μM HP-0921 was injected continuously into each sample to recalibrate for accurate mass and drift time during data processing. The ES-TOF reference mass solution kit was purchased from Agilent Technologies (Santa Clara, CA).

### Data Acquisition, Processing, and Statistical Analysis

Data acquisition and processing were performed using the Agilent MassHunter 10 software suite. Briefly, ion multiplexed data files and calibration files acquired using MassHunter Data Acquisition 10.0 software were demultiplexed using the PNNL PreProcessor v2020.03.23. The default settings for demultiplexing, moving average smoothing, saturation repair and spike removal were applied to the data. The data files were recalibrated for accurate mass and drift time using the AgtTofReprocessUi and the IM-MS Browser 10.0, respectively. Two reference masses, *m/z* 121.050873 and 922.009798 (positive ionization mode) and *m/z* 112.985587 and 1033.988109 (negative ionization mode) were used for recalibration.

Molecular features were extracted in Mass Profiler 10.0 with a retention time tolerance of ± 0.3 min, drift time tolerance of ± 1.5% and accurate mass tolerance of ± (5 ppm + 2 mDa). The raw multiplexed data, the reconstructed demultiplexed data and Mass Profiler feature lists (.cef files) were used in the High Resolution Demultiplexer (HRdm) 1.0 beta v41 for further peak deconvolution (May et al., 2020). Molecular features were re-extracted from HRdm files using Mass Profiler 10.0 and annotated using accurate mass and CCS values with the McLean CCS Compendium PCDL (version 20191101; Picache et al., 2019). A CCS value tolerance of ± 1% was applied, and the positive ion species (M+H)^+^, (M+Na)^+^, (M+NH_4_)^+^, and the negative ion species (M-H)^–^ and (M+CH_3_COO)^–^ were searched.

Multivariate statistical analysis and pathway enrichment analysis were performed using the MetaboAnalyst 5.0 web-based platform (Chong and Xia, 2020). The data were log-transformed and auto-scaled. To get an overview of the entire dataset, both annotated and unannotated molecular features were used to generate PLS-DA plots and heatmaps. To perform pathway enrichment analysis, only the annotated features without the relative peak intensities was used. For more detailed analysis of the altered pathways, the list of annotated compounds with their relative intensities was submitted to the pathway analysis tool, log-transformed, auto-scaled and examined against the *Danio rerio* KEGG pathway library, using global test and relative betweenness centrality methods. The data was visualized as scatter plots. For the pathways identified to be significantly altered between the study groups, the dataset was manually screened to identify the intermediates that were not recognized by MetaboAnalyst 5.0 online platform owing to synonymous annotations. Box and whisker plots for all the identified metabolites were retrieved from the feature details view following PLS-DA analysis on MetaboAnalyst 5.0 online platform, and the normalized values are presented.

The eumelanin biosynthesis pathway intermediates were searched for in the dataset using accurate mass values. The only precursor identified was 5,6-dihydroxyindole-2-carboxylic acid with an accurate mass of *m/z* 193.03751. To calculate ATP to ADP ratio, the peak intensity values in the annotated dataset were used and relative abundance is presented. The 260 annotated features were classified into compound classes using ClassyFire, the web-based application for automated structural classification of chemical entities (Djoumbou Feunang et al., 2016). The output file was manually curated to unambiguously assign a compound class to the metabolite. The data is presented as a two-dimensional pie chart generated in excel.

## Results

### HRAS^G12V^ Expression in Basal Skin Cells Rapidly Drives Enhanced Proliferation

**FIGURE 2:**
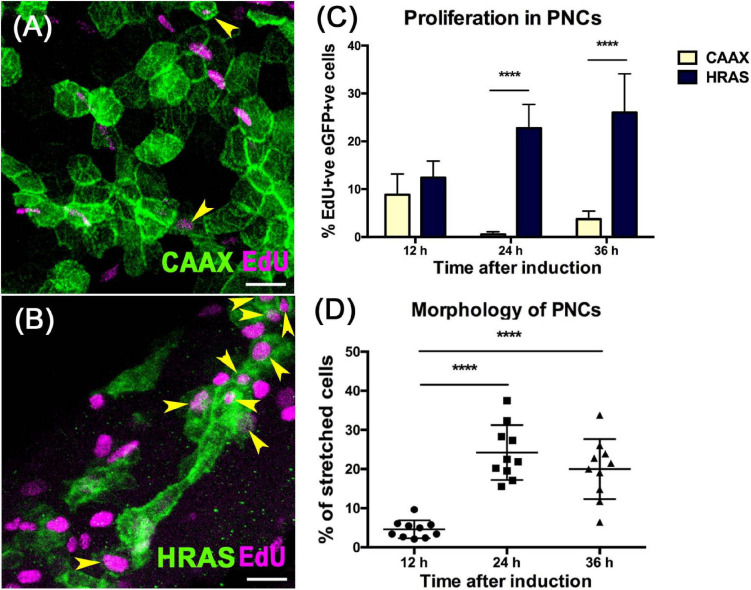
HRAS^G12V^ expressing PNCs show increased proliferation and altered morphology from 24 hpi. **(A)** Representative confocal image showing EdU labeling (Magenta) in skin cells expressing eGFPCAAX (Green) at 24 hpi. **(B)** Representative confocal image showing EdU labeling (Magenta) in skin cells expressing eGFPHRAS^G12V^(Green) at 24 hpi. Yellow arrows indicate EdU^+ve^ eGFP^+ve^ cells, scale bar = 20 μm. **(C)** Graph showing increased proliferation in PNCs compared to eGFPCAAX expressing healthy skin cells 12 hpi (*p* = 0.057, *n* = 10); 24 hpi (*p* = 3.602e-011, *n* = 10), 36 hpi (*p* = 1,021e-07, *n* = 10), unpaired *t*-test with Holm-Sidak method for correction of multiple comparisons. **(D)** Assessing cell morphology shows that from 24 hpi, a significant proportion of PNCs have lost the normal polygonal shape and become elongated or developed long filopodia (one-way ANOVA with Tukey’s multiple comparisons test, ****p < 0.0001).

Zebrafish larval skin is a bi-layer epithelium, in which the basal layer contains the stem cell compartment that gives rise to all strata of adult skin cells (Lee et al., 2014). A krtt1c19e promoter fragment has been shown to drive specific gene expression in this basal compartment (Lee et al., 2014). To achieve precise temporal control of oncogene expression, we used a tamoxifen inducible Gal4/UAS system (Figure 1A; Ramezani et al., 2015). This system relies on the combinatorial effect of two separate elements: the zebrafish optimized version of the transcription factor Gal4, KalTA4, fused to the modified ligand-binding domain of human estrogen receptor α (ER^T2^) expressed under the control of the krtt1c19e promoter, and the Upstream Activating Sequence (UAS) controlling the expression of eGFP fused to either HRAS^G12V^ or CAAX control (van den Berg et al., 2019). We induced eGFP-HRAS^G12V^ expression in basal skin cells from 52 hpf (Figure 1A) to drive pre-neoplastic cell (PNC) development. The membrane eGFP labeling of eGFP-HRAS^G12V^ expressing PNCs allowed us to visualize morphological changes of PNCs compared with control cells (Figures 2A,B,D). At 12 hpi most of PNCs maintain their typical keratinocyte polygonal shape or slightly rounded but around 4.6% of PNCs appear to lose their polygonal shape and became more stretched out, by 24 hpi the morphology of PNCs diversifies further with 24% acquiring a more elongated or irregular shape or developing filopodia-like structures compared with polygonal shaped control eGFP-CAAX expressing cells (Figures 2B,D and Supplementary Figure 4). EdU incorporation experiments indicate that PNCs acquire an enhanced proliferation capacity at 24 hpi (22.75 ± 4.95%) compared with CAAX controls (0.55 ± 0.55%) and 12hpi PNC (12.41 ± 3.46) (Figures 2B,C and Supplementary Data File 1). The cell morphology changes and enhanced proliferation rate of PNCs is maintained at 36 hpi (Figures 2C,D). Since enhanced proliferation and clonal expansion are key features of pre-neoplastic development, and the initial morphological changes that might indicate the beginning of cellular state change started at 24 hpi, we decided to use larvae at 24 h post RAS oncogene induction for our untargeted metabolomics study to capture the early metabolic alterations in tumor initiation (Figure 1).

### Whole-Mount Surface Extraction Coupled With LC-MS-IMS Allows a Comprehensive Survey of Metabolites From Zebrafish Larval Skin Tissue

**FIGURE 3:**
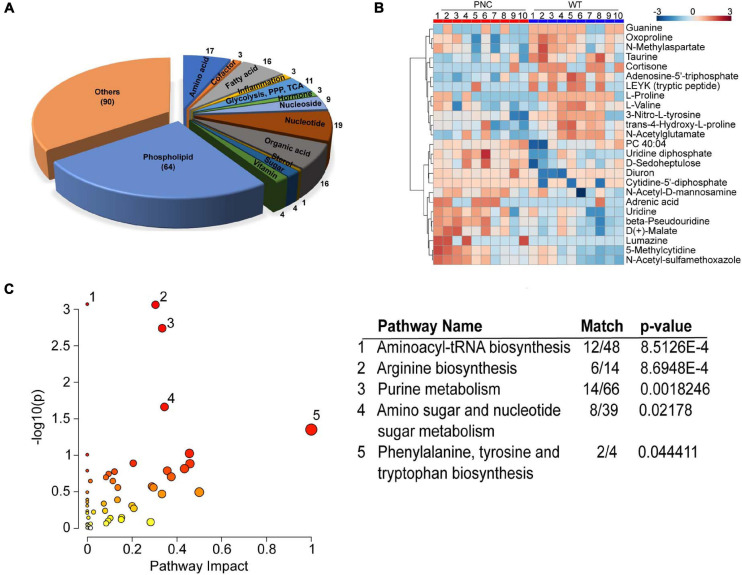
Metabolites extracted from zebrafish larval skin tissue. **(A)** Two-dimensional pie chart showing the distribution of 260 annotated metabolites assigned into compound classes using ClassyFire. The metabolites in each class are represented in the wedges and indicated with a number. **(B)** Heatmap generated using MetaboAnalyst 5.0 online platform showing the preneoplastic cell (PNC) and control (WT) groups for *N* = 10. The normalized relative abundance is presented in a gradient from blue (low) to red (high). **(C)** Scatter plot of *Danio rerio* KEGG metabolic pathways identified in the metabolomics dataset. The *p*-values from the pathway enrichment analysis are shown in darker color indicating more significant changes within a pathway, while the pathway impact values from the pathway topology analysis are depicted by the size of the node. Significantly (*p* < 0.05) impacted pathways are numbered and tabulated: 1, Aminoacyl-tRNA biosynthesis; 2, Arginine biosynthesis; 3, Purine metabolism; 4, Amino sugar and nucleotide sugar metabolism; 5, Phenylalanine, tyrosine and tryptophan biosynthesis. The match column shows the number of metabolites identified within the KEGG pathway.

In order to survey metabolic perturbations in the HRAS^G12V^ induced skin pre-neoplastic lesions, we developed a whole-mount metabolite extraction protocol (Figures 1B,C) using an ice-cold homogenous mixture of chloroform/methanol/water (1:3:1) as extraction solvent. A group of intact larvae were immersed in this solvent mixture and incubated for 40 min allowing unbiased extraction of both polar and apolar metabolites from the larval skin tissue. Pigment extracted from skin could be seen dissolved in the solvent resulting in a yellowish solution and leaving intact larvae with a pale color (Supplementary Figure 3). The metabolic extract was subjected to chromatographic separation using zwitterionic-phase hydrophilic interaction chromatography (ZIC-pHILIC) coupled to MS-IMS detection in positive and negative ionization modes (Figure 1D). By using retention time, *m/z* and ion mobility drift time and CCS values, a total of 7,094 molecular features were obtained in positive and negative ionization modes combined. Multivariate statistical analysis using partial least squares-discriminant analysis (PLS-DA) on all the molecular features, separated the pre-neoplastic group from the wild-type sample group (Supplementary Figure 1A). Using the McLean CCS Compendium library consisting of 1,446 metabolites, 1,376 (19.4%) molecular features corresponding to 260 metabolites were annotated in the dataset across the two sample groups, preneoplastic vs. healthy larval skin (Figure 1E). Of these, 170 (65.4%) metabolites were unambiguously classified into 13 compound classes, with an overrepresentation (64 of 260 metabolites, 24.6%) of phospholipids, including the skin lipids phosphatidylcholine (PC), phosphatidylethanolamine (PE), phosphatidylserine (PS), and sphingomyelin (SM) (Figure 3A and Supplementary Data File 2). Additionally, the eumelanin precursor 5,6-dihydroxyindole-2-carboxylic acid was also detected (using accurate mass search) in the extracts of both sample groups at similar level (Supplementary Figure 1B and Supplementary Data File 2), demonstrating efficient and consistent metabolite extraction from the larval skin tissue across samples. To verify that our protocol is restricted to extracting metabolites from skin tissue only, we specifically queried the metabolomics dataset for lactate (using accurate mass search), which is one of the most enriched metabolites in muscle tissue just underneath the skin. Interestingly, we could not detect lactate in our samples, suggesting that our extraction was likely to be limited to skin tissue.

Further analysis of the 260 annotated metabolites showed differences in their relative abundance across the sample groups and the expected biological variation across the dataset (Figures 1F, 3B), emphasizing the need for multiple biological replicates to capture the true variation in the zebrafish larval skin model. A pathway enrichment analysis of the 260 annotated metabolites identified the representation of five statistically significant (*p* < 0.05) pathways corresponding to purine, amino acid and aminoacyl-tRNA metabolism in the dataset (Figure 3C).

### Comparative Analysis Identifies Metabolic Pathways Altered in Preneoplastic Development 24 h Post-HRAS^G12V^ Induction

**FIGURE 4:**
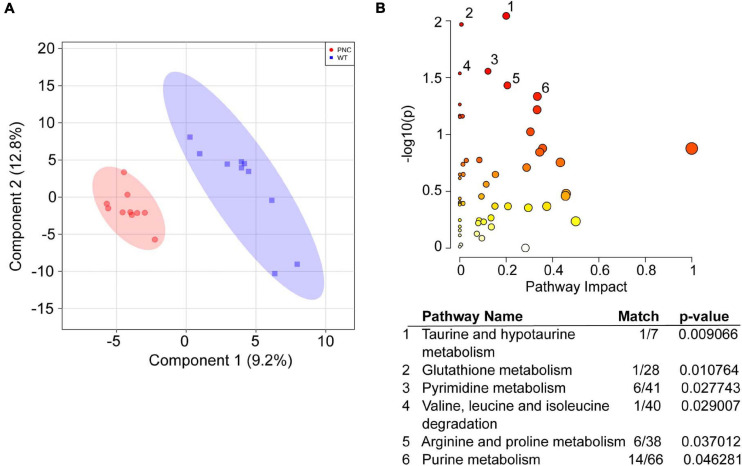
Metabolic pathways significantly altered upon HRAS^G12V^ induction in PNCs. **(A)** Partial Least Squares—Discriminant Analysis (PLS-DA) score plot shows separation of the preneoplastic cell (PNC) and control (WT) groups. The first and second components explain 9.2 and 12.8% of the variation, respectively. Different colors and shapes correspond to PNC, red circle and WT, blue square. **(B)** Scatter plot of *Danio rerio* KEGG metabolic pathways significantly altered upon HRAS^G12V^ induction. The *p*-values from the pathway enrichment analysis are shown in darker color indicating more significant changes within a pathway, while the pathway impact values from the pathway topology analysis are depicted by the size of the node. Significantly (*p* < 0.05) impacted pathways are numbered and tabulated: 1, Taurine and hypotaurine metabolism; 2, Glutathione metabolism; 3, Pyrimidine metabolism; 4, Valine, leucine and isoleucine degradation; 5, Arginine and proline metabolism; 6, Purine metabolism. The match values indicate the number of metabolites identified within the KEGG pathway.

To identify the metabolic pathways altered upon HRAS^G12V^ induction, the 260 annotated metabolites in the two sample groups were carried forward for multivariate statistical analysis using the MetaboAnalyst 5.0 platform. The PLS-DA analysis for WT and PNC samples clearly separated the two groups with principal components 1 and 2 explaining 9.2 and 12.8% of the variation, respectively (Figure 4A). A pathway enrichment analysis identified six metabolic pathways, including pyrimidine, purine and amino acid metabolism to be significantly (*p*-value < 0.05) altered across the sample groups (Figure 4B). Given the higher number of metabolites identified within the pathways for pyrimidine (6/41), arginine and proline (6/38) and purine (14/66) metabolism (Figure 4B, table), we focused on analyzing the possible role of these metabolic networks in preneoplastic cell development.

### Altered Pyrimidine Metabolism Detected Upon PNC Development

**FIGURE 5:**
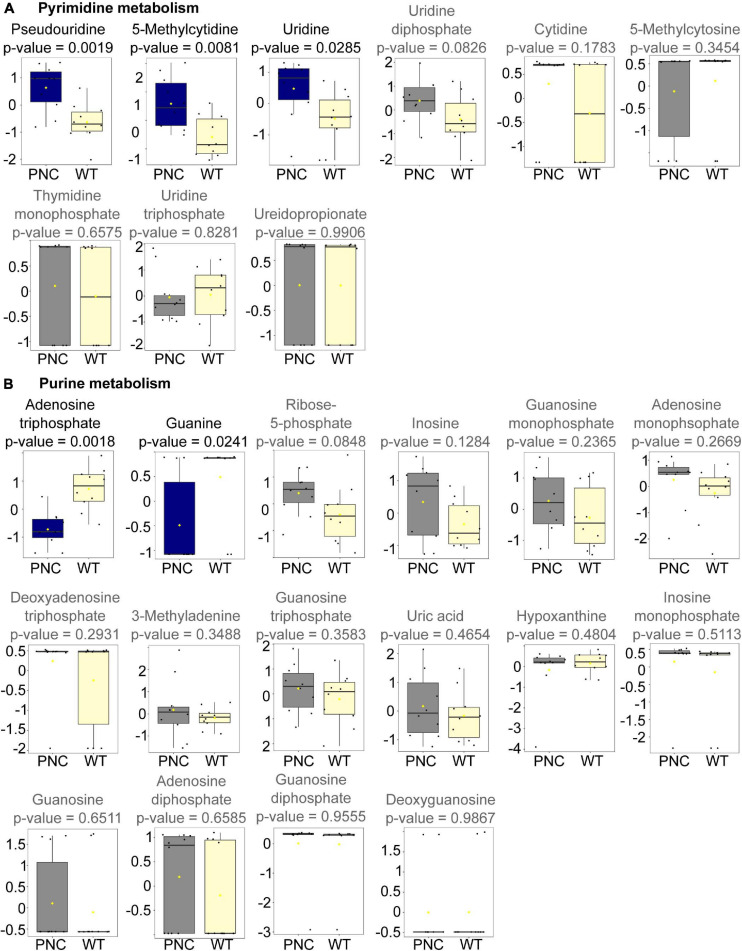
Metabolites significantly altered upon HRAS^G12V^ induction in PNCs. Box and whisker plots generated using MetaboAnalyst 5.0 online platform for individual metabolites in pyrimidine **(A)** and purine **(B)** metabolism identified in our dataset are presented with the statistically significant (*p* < 0.05) altered metabolites presented in blue and the non-significantly altered metabolites presented in gray. The boxplots represent median ± interquartile range of log transformed and auto-scaled intensities for preneoplastic cell (PNC) and control (WT) groups (*N* = 10).

Nine metabolites involved in pyrimidine metabolism were annotated in our dataset (Figure 5A). Significant increase in the levels of 5-methylcytidine (*p* = 0.0081), uridine (*p* = 0.0285), and its isomer pseudouridine (*p* = 0.0019) were observed, suggesting a potential role for these metabolites in the development of preneoplastic lesions. Uridine is a key substrate for generating uridine monophosphate (UMP, not annotated in our dataset) by uridine-cytidine kinase in the salvage pathway (Supplementary Figure 2; Connolly and Duley, 1999). UMP is the key entry molecule for generating the full spectrum of pyrimidine nucleotides and nucleic acids (Supplementary Figure 2; Connolly and Duley, 1999; Kanehisa and Goto, 2000). Although UMP is not annotated in our dataset, we detected uridine diphosphate (UDP, *p* = 0.0826) and uridine triphosphate (UTP, = 0.8281), both of which were present at similar levels in the PNC and WT groups (Figure 5A and Supplementary Figure 2). This suggests the ability of PNC to maintain a pyrimidine nucleotide pool under high proliferative demand through a salvage pathway.

The conversion of uridine to 3-ureidopropionate is an important step for pyrimidine degradation (Kanehisa and Goto, 2000). Our data shows similar levels of 3-ureidopropionate (*p* = 0.9906) in PNC and WT groups (Figure 5A and Supplementary Figure 2), suggesting similar levels of pyrimidine degradation. Additionally, cytidine (*p* = 0.1783), 5-methycytosine (*p* = 0.3454), and thymidine monophosphate (TMP, *p* = 0.6575) were also annotated in the dataset, and no significant changes in their relative abundance was observed between the sample groups (Figure 5A and Supplementary Figure 2).

### Altered Purine Metabolism Detected Upon PNC Development

Unlike the detectable higher abundance for some pyrimidine metabolites, a reduced relative abundance of guanine (*p* = 0.0241) and adenosine triphosphate (ATP, *p* = 0.0018) was detected in the PNC samples compared to the control group (Figure 5B). Fourteen additional purine metabolites were annotated in the dataset with no significant (*p* > 0.05) differences in their relative abundance between the sample groups, although a slight trend of increase could be visualized for three metabolites (ribose-5-phosphate, adenosine monophosphate and inosine) in the PNC samples (Figure 5B and Supplementary Figure 2).

In addition to ATP, the energy charge metabolites adenosine monophosphate (AMP, *p* = 0.2669) and adenosine diphosphate (ADP, *p* = 0.6585) were also annotated in our dataset. Since reduced ATP to ADP ratio has been reported to favor enhanced glycolysis in proliferating cells (Maldonado and Lemasters, 2014), we determined this ratio in our study. We calculated the ATP to ADP ratio using the raw peak intensity values, and indeed found a trend of reduced ATP/ADP ratio (*p* = 0.084) in the PNC group compared to WT group (Supplementary Figure 1C), which likely correlates with enhanced proliferation in preneoplastic lesions.

### Changes in Amino Acid Metabolism Upon PNC Development

**FIGURE 6:**
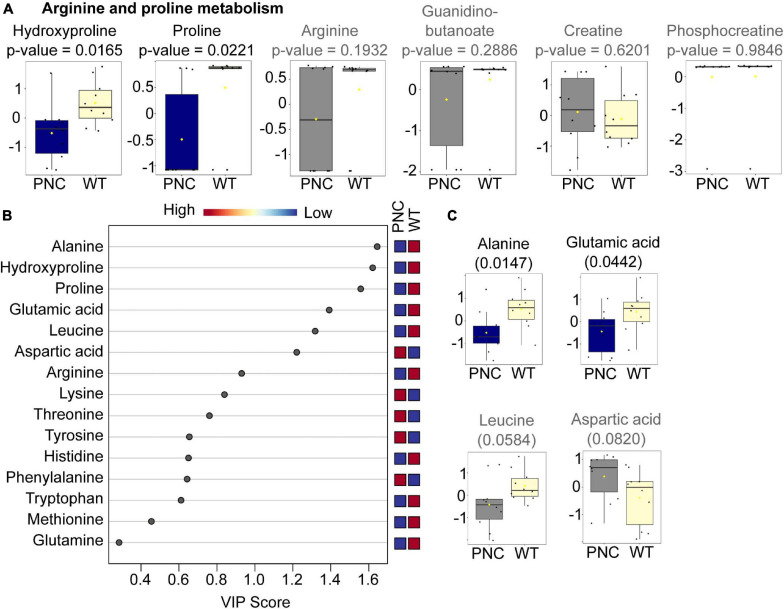
Amino acids significantly altered upon HRAS^G12V^ induction in PNCs. **(A)** Box and whisker plots generated using MetaboAnalyst 5.0 online platform for arginine and proline metabolism, identified using pathway enrichment analysis. **(B)** Variable importance in projection (VIP) plot generated using MetaboAnalyst 5.0 online platform for the 15 amino acids identified in our dataset. The relative abundance of each amino acid in the preneoplastic cell (PNC) and control (WT) groups are presented as colored boxes ranging from blue (low) to red (high). The average for *N* = 10 is presented. **(C)** Box and whisker plots for the four amino acids with a VIP cut-off score of 1, except hydroxyproline and proline presented in **(A)**. The altered metabolites with statistical significance (*p* < 0.05) are presented in blue and the non-significant metabolites in gray. The boxplots represent median ± interquartile range of log transformed and auto-scaled intensities for preneoplastic cell (PNC) and control (WT) groups (*N* = 10).

In arginine and proline metabolism, significantly lower relative abundance of hydroxyproline (*p* = 0.0165) and proline (*p* = 0.0221) was observed in PNC samples (Figure 6A). In addition, four other metabolites were detected in our dataset, of which arginine (*p* = 0.1932) and guanidinobutanoate (*p* = 0.2886) show a trend of lower abundance (Figure 6A), while creatine and phosphocreatine showed no differences in their relative abundance, compared to the WT group. Given these differences and because other amino acid metabolic pathways were among the pathways identified to be significantly altered (Figure 4B), we analyzed the relative abundance of all amino acids in our dataset. Of the 20 amino acids, 15 were detected in our untargeted analysis (Supplementary Data File 2). Ten of those amino acids were relatively lower in PNC group (Figure 6B). Using PLS-DA analysis and a variable importance in projection (VIP) cut-off score of 1, six amino acids were identified to be changed of which alanine (*p* = 0.0147), hydroxyproline (0.0165), proline (0.0221), and glutamic acid (0.0442) were significantly lower in the PNC group (Figures 6A,C). Additionally, leucine was found to have a trend of lower abundance (*p* = 0.0584) and aspartic acid higher (0.0820) in the PNC group (Figure 6C), suggesting alterations in amino acid biosynthetic pathways upon HRAS^*G12V*^ induced preneoplastic development.

### Changes in Expression of Genes Corresponding to Metabolic Enzymes Detected in PNC Development

**FIGURE 7:**
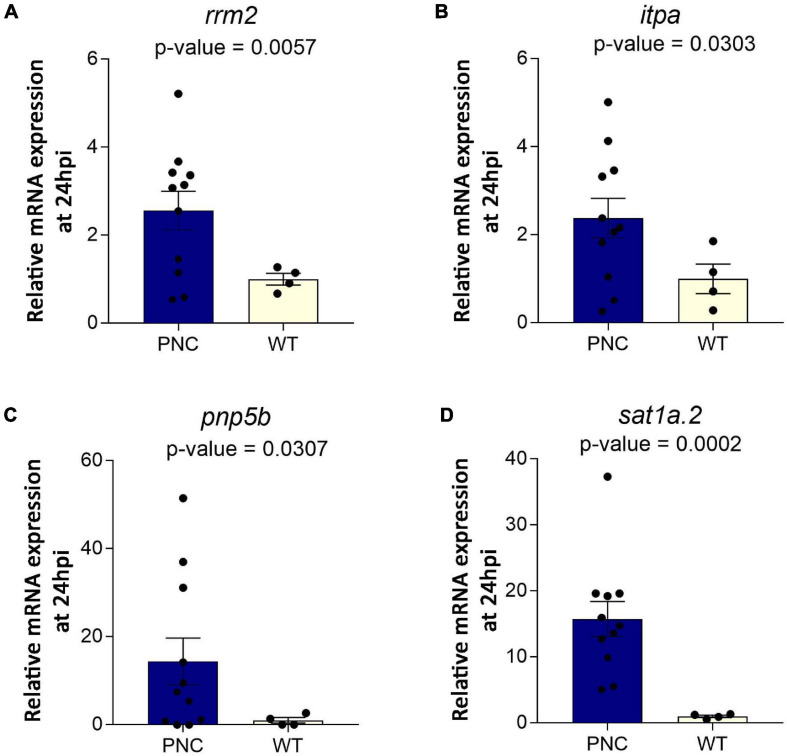
Upregulation of genes encoding metabolic enzymes at 24 h post HRAS^*G12V*^ induction in PNCs. Relative mRNA expression normalized to β-actin showing **(A)** *rrm2* (*p* = 0.0057), **(B)** *itpa* (*p* = 0.0303), **(C)** *pnp5b* (*p* = 0.0307), and **(D)** *sat1a.2* (*p* = 0.0002) expression was increased in PNCs. PNC *N* = 11, Control *N* = 4, *Welsh’s t-*test.

Many rate-limiting metabolic enzymes are known to be deregulated in cancer (Furuta et al., 2010). The changes detected in our untargeted metabolomics analysis would suggest altered activities of some of these metabolic enzymes. However, it is not clear whether these metabolic enzyme changes occur at the level of gene expression upon oncogene induction. Therefore, we determined the expression level of a handful of candidate genes implicated in cancer and found several of these to be upregulated in PNCs compared to control cells (Figure 7). Relevant to nucleotide metabolism pathways, are the genes *rrm2, itpa* and *pnp5b*, all of which were significantly overexpressed in the PNCs (Figures 7A–C). *Rrm2* encodes the ribonucleoside-diphosphate reductase subunit M2, which catalyzes the rate-limiting biosynthesis of deoxyribonucleotides from the corresponding ribonucleotides to support DNA synthesis and cell proliferation (Kittler et al., 2004; Furuta et al., 2010). Enhanced expression of *rrm2* in PNCs would support their enhanced proliferation. Inosine triphosphatase (itpa) is involved in recycling purines by converting (deoxy)inosine triphosphate [(d)ITP] to (d)IMP or xanthosine 5′-triphosphate (XTP) to XMP, which could then be recycled to contribute to DNA and RNA synthesis (Simone et al., 2013). Increased *itpa* expression in PNCs would support altered nucleotide metabolism, which we observed in our metabolomics data, although we did not detect changes in substrates or products of *itpa*, suggesting altered flux through the associated pathways rather than the accumulation of metabolites directly linked to the enzyme. *Pnp5b* encodes zebrafish purine nucleoside phosphorylase 5b, that is homologous to mammalian purine nucleoside phosphorylase, which catalyzes the reversible phosphorylation of purine nucleosides (Williams et al., 1984). Upregulation of *pnp5b* could contribute to altered levels of purine metabolites, such as guanine, hypoxanthine, guanosine and inosine, detected in our metabolomics analysis. Interestingly, we also observed increased expression of *sat1a.2* (Figure 7D), which encodes spermidine/spermine N(1)-acetyltransferase, a rate –limiting enzyme that catalyzes the N1-acetylation of spermindine and spermine (Allmeroth et al., 2021), suggesting that altered polyamine removal might occur in PNCs. However, these metabolites were not annotated in the metabolomics dataset.

## Discussion

A better understanding of the cellular and molecular changes during the earliest stages of preneoplastic development would help to elucidate the mechanisms mediating the transition from normal healthy somatic cell to abnormal tumor development, which is still an outstanding question in cancer biology (Martincorena and Campbell, 2015; Martincorena et al., 2017; Balmain, 2020).

Here, we took advantage of a tissue specific inducible gene expression system in zebrafish larval skin to gain precise temporal control of oncogenic driver gene induction (Ramezani et al., 2015; van den Berg et al., 2019). We used zebrafish larvae within 5 days post fertilization, before free feeding, when individual larvae are highly homogenous and only use yolk as their energy source for metabolism (Quinlivan and Farber, 2017). This allowed semi-quantitative analysis of their metabolome and comparison of the relative metabolite abundance between larvae with PNCs vs. their control siblings. The expression of human HRAS^G12V^ led to initiation of PNC development in the basal epithelium of zebrafish larval skin and significantly enhanced proliferation that was detected in PNCs at 24 h post HRAS^G12V^ induction. Our approach of applying untargeted metabolomics to this oncogenic HRAS mediated preneoplastic model, for the first time provides a detailed analysis of metabolic alterations at the earliest time point during oncogene induced PNC development *in vivo*. We identified six pathways to be significantly altered in the preneoplastic lesions at 24 h post HRAS^G12V^ induction, suggesting that these might play a significant role during PNC development.

Increased nucleotide metabolism, encompassing purine and pyrimidine metabolism, supports uncontrolled growth of metabolically active cells, or tumors (Di Virgilio, 2012; Wang et al., 2017; Mollick and Laín, 2020; Siddiqui and Ceppi, 2020). Significant changes in both purine and pyrimidine metabolic pathways during PNC initiation were highlighted in our study which correlate with the metabolic requirements of rapidly proliferating PNCs (Figure 5 and Supplementary Figure 2; Villa et al., 2019; Mollick and Laín, 2020). In order to gain further understanding of key genes and pathways that mediate the metabolic changes that we detected, our future work will explore single cell transcriptomic analysis of PNCs and cells in their environment. As a first glimpse into the transcriptome and to complement our metabolic studies, we have examined a handful of candidates and detected altered expression of genes (*rrm2, itpa*, and *pnp5b*) encoding metabolic enzymes involved in purine and pyrimidine metabolism. Among these genes, *rrm2* is critical in maintaining cell proliferation and has been identified as a target for cancer therapy (Kittler et al., 2004; Furuta et al., 2010). We also observed upregulation of *sat1a.2*, involved in polyamine metabolism, which can be modulated by oncogenic pathways and its deregulation is associated with some cancers (Casero et al., 2018). Increased SAT1 activity is reported to increase susceptibility to skin carcinogenesis in a transgenic mouse model (Wang et al., 2007), and more recently the upregulation of SAT1 was reported to drive tumor aggressiveness and radiation response (Brett-Morris et al., 2014; Thakur et al., 2019). The upregulation of sat1a.2 in PNCs could be a result of activated RAS signaling, which has been shown to enhance polyamine transport in cancer cells and modulate polyamine metabolism (Basu Roy et al., 2008). Further experiments are required to firmly establish whether HRAS^G12V^ mediated signaling directly regulates the expression of these metabolic genes.

Interestingly, in the preneoplastic lesions we detected increased levels of 5-methylcytidine, which is an important epitranscriptomic marker that is mostly found in tRNA and rRNA but also less frequently in mRNA (Squires et al., 2012; Trixl and Lusser, 2019). Elevated levels of methylated purines detected in urine of cancer patients was due to accelerated tRNA turn over in tumor tissue (Mandel et al., 1966; Borek et al., 1977). This could be one explanation as to why we detected increased 5-methylcytidine, suggesting higher turnover rate of tRNA in PNC tissue. In addition, modified nucleosides cannot be recycled by scavenger enzymes, therefore elevated 5-methylcytidine might indicate altered post-transcriptional modification of RNA, which plays a regulatory role in protein translation (Peer et al., 2019). The NOP2/Sun RNA methyltransferase family member 2 (NSUN2) catalyzes formation of 5-methylcytidine (m5C) in RNAs (Brzezicha et al., 2006; Blanco et al., 2011). Although the functional role of RNA cytosine-5 methylation is not fully understood in cancer development, expression of NSUN2 is elevated in tumors of multiple tissue origin suggesting a fundamental link to tumor progression (Frye and Watt, 2006; Okamoto et al., 2012; Yi et al., 2017). Similarly, cytosine DNA methyltransferase Dnmt2 is frequently found to be mis-regulated or mutated in tumors (Elhardt et al., 2015; Wang et al., 2020). RNA cytosine-5 methylation has also been shown to be important for normal development and cellular stress responses, in addition to cancer (Blanco et al., 2011; Metodiev et al., 2014; Popis et al., 2016; Gkatza et al., 2019; Xiang et al., 2020). Our data suggests that alterations in post-transcriptional modification of RNA might be an early event during oncogene induced preneoplastic development. This warrants further study to confirm these changes and to address their functional role during PNC development.

Changes in amino acid metabolism have a broad impact on cellular function and physiology (Brosnan, 2001; Wu, 2009; Kelly and Pearce, 2020). Many of such changes have been described in cancer (Phillips et al., 2013; Tardito et al., 2015; Choi and Coloff, 2019; Sivanand and Vander Heiden, 2020; Vettore et al., 2020). We detected significant changes in amino acid metabolism within 24 h of oncogenic RAS expression, highlighting the sensitivity of amino acid metabolism in response to cellular perturbation. However, further studies are needed to elucidate the mechanisms that might mediate these changes and the functional consequence of such metabolic alterations. It is worth noting that polyamine metabolism was found to be perturbed in human oral cavity squamous cell carcinoma (Hsu et al., 2019). Although we detected changes in gene expression of sat1a.2, a key enzyme in polyamine metabolism, our metabolomics data did not annotate relevant metabolites. In the same study, phenylalanine and threonine were found to be up-regulated in tumor tissue compared to adjacent normal tissues (Hsu et al., 2019) and these amino acids although not significant, also showed a trend of increased abundance in our PNC samples. This suggests that similar metabolic changes could persist during the process of cancer development, although cancer cells would need to alter their metabolism to adapt to changes in their environment during the process of cancer development. There is still more work to be done in improving technical aspects of our procedure and data annotation, which is one of the major bottlenecks in untargeted metabolomics.

In conclusion, we have established a novel workflow for metabolite extraction and untargeted metabolomics analysis to identify and semi-quantify alterations of metabolites in preneoplastic lesions in zebrafish larval skin. The untargeted metabolomics analysis allows an unbiased identification of changes in metabolic pathways. Our proof of principle analysis using the oncogenic human HRAS^G12V^ induced PNC model has identified altered pathways that warrant further study. Our model and protocol can be expanded to analyze metabolic changes in PNCs that are triggered by different oncogenes, since different downstream signaling pathways from different oncogenes will likely trigger different metabolic changes. This metabolomic analysis could also be further combined with transcriptomic and/or proteomic analyses of PNCs to identify mechanisms involved in the crosstalk between oncogene modulated signaling and changes in metabolism.

## Data Availability Statement

The original contributions presented in the study are included in the article/Supplementary Material, further inquiries can be directed to the corresponding author/s.

## Ethics Statement

The animal study was reviewed and approved by the Local Ethical Committee at The University of Edinburgh.

## Author Contributions

HM, TM, and YF designed the project. HM, TM, JAJ, and AE performed the experiments. HM, TM, JAJ, EGP, and AE analyzed the data. HM, TM, JAJ, EGP, and YF constructed the figures. YF and TM wrote the manuscript with contribution from all authors. YF conceived the idea and acquired funding.

## Conflict of Interest

The authors declare that the research was conducted in the absence of any commercial or financial relationships that could be construed as a potential conflict of interest.

## Publisher’s Note

All claims expressed in this article are solely those of the authors and do not necessarily represent those of their affiliated organizations, or those of the publisher, the editors and the reviewers. Any product that may be evaluated in this article, or claim that may be made by its manufacturer, is not guaranteed or endorsed by the publisher.

## Supplementary Material

Supplementary Figure 1 Overview of the metabolomics dataset using annotated and unannotated metabolic features. **(A)** Partial Least Squares—Discriminant Analysis (PLS-DA) score plot shows separation of the preneoplastic cell (PNC), control (WT) and pooled quality control (QC) groups. The first and second components explain 5.4 and 5.8% of the variation, respectively. Different colors and shapes correspond to PNC, red circle; WT, blue square; QC, orange triangle. The pooled quality control sample was generated by mixing equal volumes of each sample, to obtain a representative sample for the experiment. QC data was acquired by injecting one sample after every five experiment samples. **(B)** The eumelanin precursor 5,6-dihydroxyindole-2-carboxylic acid was identified using accurate mass (*m/z* 193.03751) in the PNC and WT groups. Ion abundance obtained from the instrument is presented with error bars corresponding to standard deviation (*N* = 10). **(C)** The ratio of ATP to ADP in the preneoplastic cell (PNC) and control (WT) groups is presented with error bars corresponding to standard deviation (*N* = 10). ADP, adenosine diphosphate; ATP, adenosine triphosphate.

Supplementary Figure 2 Pyrimidine and purine metabolic pathways and the crosstalk between the pathway intermediates is presented. The metabolic intermediates identified in our dataset as shown as box and whisker plots with statistically significant metabolites presented in blue and non-significant metabolites in gray. The boxplots represent median ± interquartile range of log transformed and auto-scaled intensities for preneoplastic cell (PNC) and control (WT) groups (*N* = 10). ADP, adenosine diphosphate; AICAR, 5-aminoimidazole-4-carboxamide ribonucleotide; AMP, adenosine monophosphate; ATP, adenosine triphosphate; CDP, cytosine diphosphate; CTP, cytosine triphosphate; dATP, deoxyadenosine triphosphate; dCDP, deoxycytosine diphosphate; dCMP, deoxycytosine monophosphate; dUMP, deoxyuridine monophosphate; GDP, guanosine diphosphate; GMP, guanosine monophosphate; GTP, guanosine triphosphate; IMP, inosine monophosphate; PRPP, phosphoribosyl pyrophosphate; S-AMP, succinyl-AMP; TMP, thymidine monophosphate; UDP, uridine diphosphate; UMP, uridine monophosphate; UTP, uridine triphosphate; XMP, xanthine monophosphate.

Supplementary Figure 3 Representative image of metabolites extracted into solution.

Supplementary Figure 4 Representative images showing cell morphology changes of PNCs that generated the quantification for Figure 2D. **(A,B)** Images showing eGFPCAAX expressing cells at 24 and 36 hpi. **(C,C’)** Images showing PNCs at 12 hpi. **(D,D’)** Images showing PNCs at 24 hpi. **(D,D’)** Images showing PNCs at 36 hpi. Cell shape is determined by membrane eGFP signal, magenta showing EdU labeled nuclei that indicate active proliferation. Dotted white lines mark edge of fish body; scale bar = 20 μm.

Supplementary Data File 1 EdU incorporation data containing the number of eGFPHRAS^G12V^ positive cells (PNCs), eGFPCAAX positive wild type skin cells, and the number of EdU positive cells among these in the images obtained from each larvae, together with the number of PNCs with an elongated morphology or filopodia.

Supplementary Data File 2 Raw output file containing the annotated metabolites and their peak intensities in each sample. The ion species identified, accurate mass and CCS value in the database are also provided.
