# Supplementary figures and images for: Metabolic Alterations in Preneoplastic Development Revealed by Untargeted Metabolomic Analysis
Source: Front Cell Dev Biol. 2021 Aug 3;9:684036. doi: 10.3389/fcell.2021.684036 (PMC8369915; doi:10.3389/fcell.2021.684036)

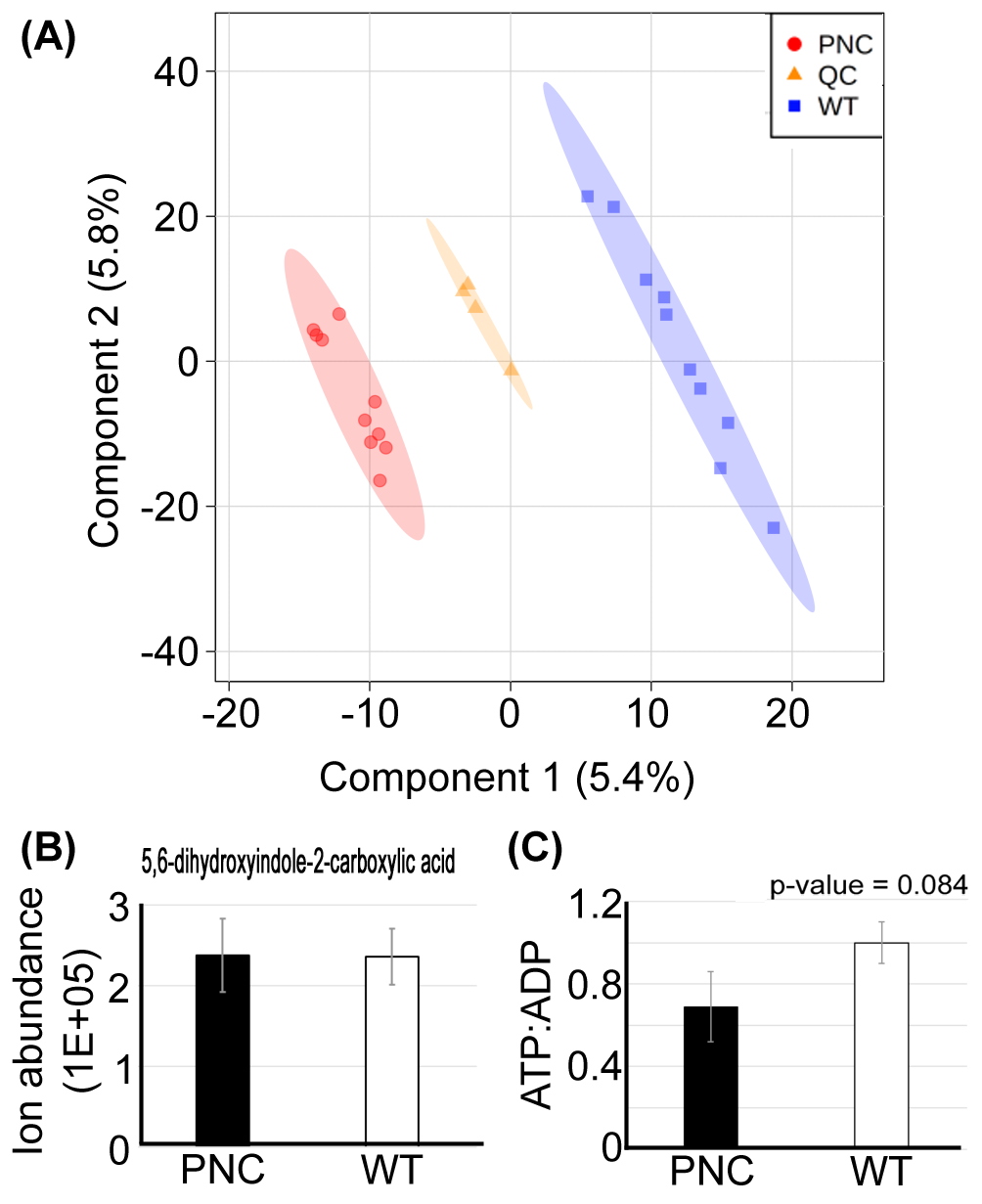

Supplement: Supplementary Figure 1 — Overview of the metabolomics dataset using annotated and unannotated metabolic features. (A) Partial Least Squares—Discriminant Analysis (PLS-DA) score plot shows separation of the preneoplastic cell (PNC), control (WT) and pooled quality control (QC) groups. The first and second components explain 5.4 and 5.8% of the variation, respectively. Different colors and shapes correspond to PNC, red circle; WT, blue square; QC, orange triangle. The pooled quality control sample was generated by mixing equal volumes of each sample, to obtain a representative sample for the experiment. QC data was acquired by injecting one sample after every five experiment samples. (B) The eumelanin precursor 5,6-dihydroxyindole-2-carboxylic acid was identified using accurate mass (m/z 193.03751) in the PNC and WT groups. Ion abundance obtained from the instrument is presented with error bars corresponding to standard deviation (N = 10). (C) The ratio of ATP to ADP in the preneoplastic cell (PNC) and control (WT) groups is presented with error bars corresponding to standard deviation (N = 10). ADP, adenosine diphosphate; ATP, adenosine triphosphate. [file Image_1.TIF]

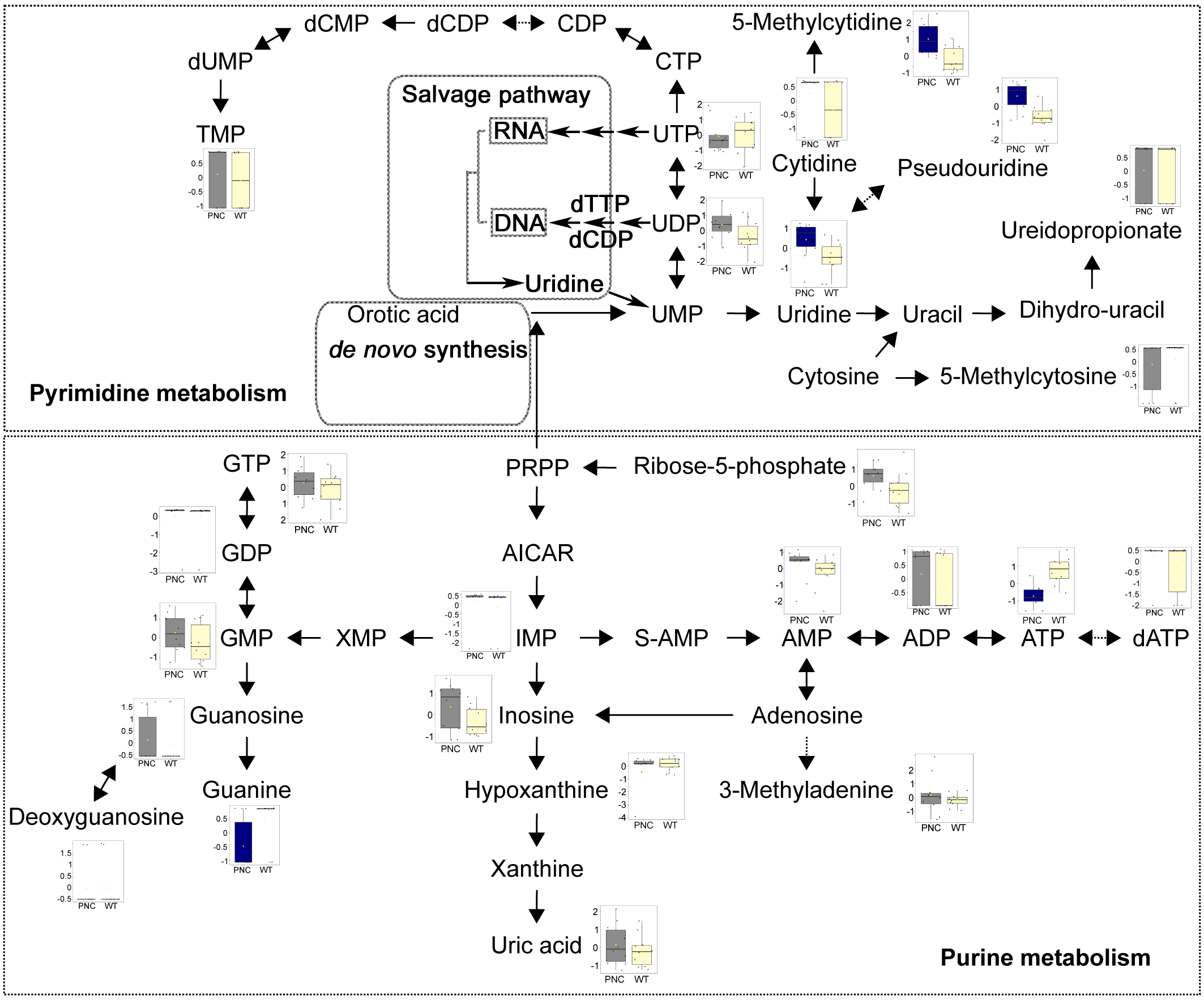

Supplement: Supplementary Figure 2 — Pyrimidine and purine metabolic pathways and the crosstalk between the pathway intermediates is presented. The metabolic intermediates identified in our dataset as shown as box and whisker plots with statistically significant metabolites presented in blue and non-significant metabolites in gray. The boxplots represent median ± interquartile range of log transformed and auto-scaled intensities for preneoplastic cell (PNC) and control (WT) groups (N = 10). ADP, adenosine diphosphate; AICAR, 5-aminoimidazole-4-carboxamide ribonucleotide; AMP, adenosine monophosphate; ATP, adenosine triphosphate; CDP, cytosine diphosphate; CTP, cytosine triphosphate; dATP, deoxyadenosine triphosphate; dCDP, deoxycytosine diphosphate; dCMP, deoxycytosine monophosphate; dUMP, deoxyuridine monophosphate; GDP, guanosine diphosphate; GMP, guanosine monophosphate; GTP, guanosine triphosphate; IMP, inosine monophosphate; PRPP, phosphoribosyl pyrophosphate; S-AMP, succinyl-AMP; TMP, thymidine monophosphate; UDP, uridine diphosphate; UMP, uridine monophosphate; UTP, uridine triphosphate; XMP, xanthine monophosphate. [file Image_2.TIF]

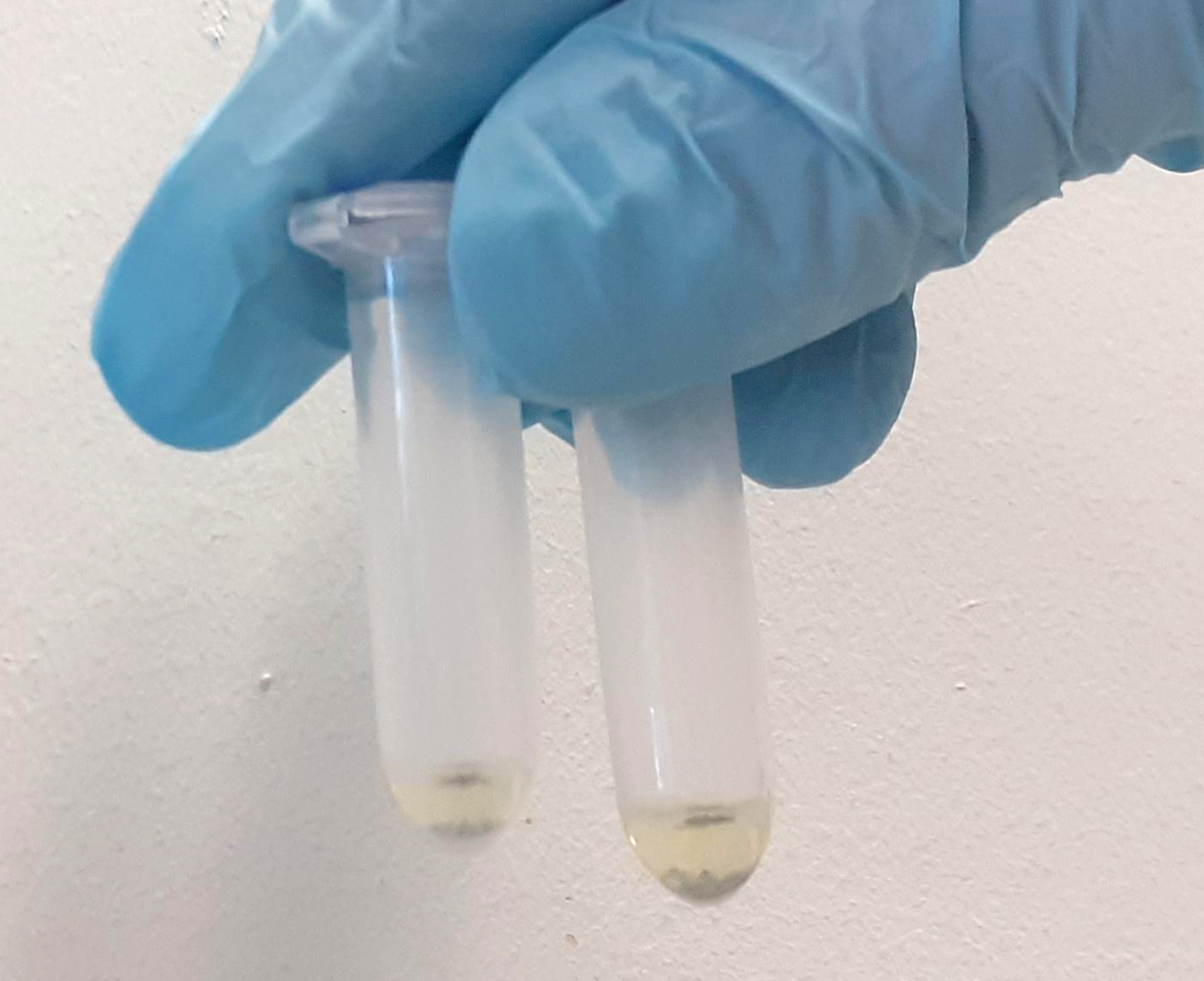

Supplement: Supplementary Figure 3 — Representative image of metabolites extracted into solution. [file Image_3.TIF]

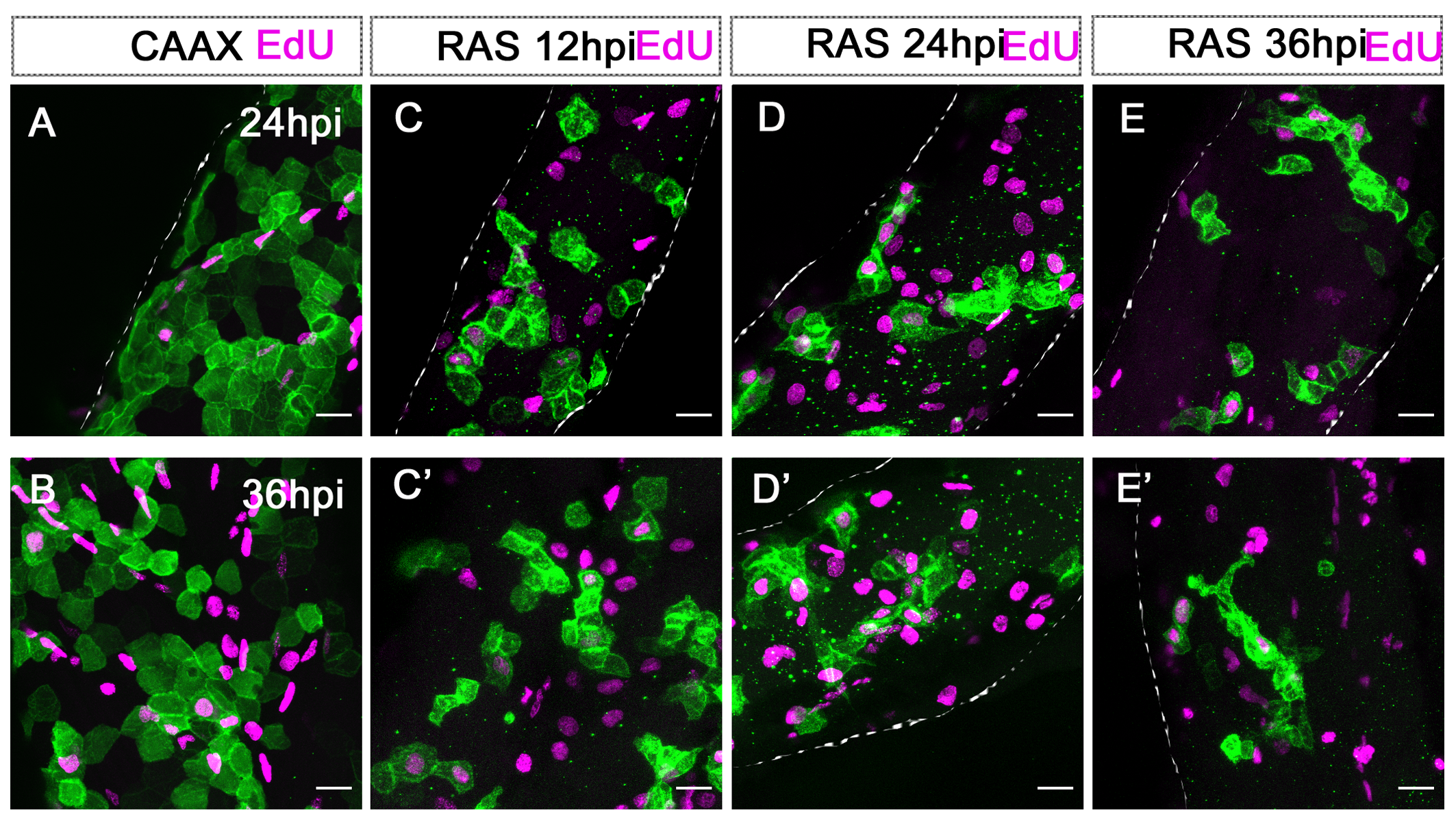

Supplement: Supplementary Figure 4 — Representative images showing cell morphology changes of PNCs that generated the quantification for Figure 2D. (A,B) Images showing eGFPCAAX expressing cells at 24 and 36 hpi. (C,C’) Images showing PNCs at 12 hpi. (D,D’) Images showing PNCs at 24 hpi. (D,D’) Images showing PNCs at 36 hpi. Cell shape is determined by membrane eGFP signal, magenta showing EdU labeled nuclei that indicate active proliferation. Dotted white lines mark edge of fish body; scale bar = 20 μm. [file Image_4.TIF]
