# Supplementary material for: Metabolic Alterations in Preneoplastic Development Revealed by Untargeted Metabolomic Analysis
Source: Front Cell Dev Biol. 2021 Aug 3;9:684036. doi: 10.3389/fcell.2021.684036 (PMC8369915; doi:10.3389/fcell.2021.684036)
Supplement: Supplementary Data File 1 — EdU incorporation data containing the number of eGFPHRASG12V positive cells (PNCs), eGFPCAAX positive wild type skin cells, and the number of EdU positive cells among these in the images obtained from each larvae, together with the number of PNCs with an elongated morphology or filopodia. [file Data_Sheet_1.PDF]

Supplementary Data File 1. Cell proliferation and morphology in PNCs and control cells

K19;UAS:eGFP-CAAX

Time after induction		eGFP+ve cells	EdU+ve eGFP+ve cells	% of EdU+ve eGFP+ve cells
12 h	Fish1	168	20	11.90
	Fish2	235	16	6.81
	Fish3	187	25	13.37
	Fish4	224	9	4.02
	Fish5	220	16	7.27
	Fish6	128	7	5.47
	Fish7	194	12	6.19
	Fish8	203	36	17.73
	Fish9	170	10	5.88
	Fish10	201	20	9.95
24 h	Fish1	196	1	0.51
	Fish2	188	0	0.00
	Fish3	227	2	0.88
	Fish4	128	0	0.00
	Fish5	211	2	0.95
	Fish6	117	0	0.00
	Fish7	186	3	1.61
	Fish8	148	0	0.00
	Fish9	227	2	0.88
	Fish10	159	1	0.63
36 h	Fish1	153	6	3.92
	Fish2	139	6	4.32
	Fish3	148	5	3.38
	Fish4	151	6	3.97
	Fish5	115	5	4.35
	Fish6	146	10	6.85
	Fish7	160	3	1.88
	Fish8	158	5	3.16
	Fish9	201	10	4.98
	Fish10	172	1	0.58

K19;UAS:eGFP-HRAS^{G12V}

Time after induction		PNCs	EdU+ve PNCs	% of EdU+ve PNCs	Stretched PNCs	% of strectched PNCs
12 h	Fish1	73	11	15.07	4	5.48
	Fish2	140	26	18.57	7	5.00
	Fish3	118	18	15.25	4	3.39
	Fish4	142	10	7.04	3	2.11
	Fish5	88	8	9.09	3	3.41
	Fish6	105	11	10.48	8	9.64
	Fish7	104	10	9.62	6	5.77
	Fish8	66	9	13.64	4	6.06
	Fish9	187	22	11.76	5	2.67
	Fish10	169	23	13.61	4	2.37
24 h	Fish1	173	32	18.50	56	32.37
	Fish2	169	46	27.22	38	22.49
	Fish3	123	30	24.39	24	19.51
	Fish4	150	25	16.67	41	27.33
	Fish5	219	41	18.72	62	28.31
	Fish6	76	17	22.37	13	17.11
	Fish7	120	21	17.50	45	37.50
	Fish8	198	54	27.27	40	20.20
	Fish9	103	24	23.30	16	15.53
	Fish10	247	78	31.58	54	21.86
36 h	Fish1	157	16	10.19	53	33.76
	Fish2	162	36	22.22	19	11.73
	Fish3	115	32	27.83	17	14.78
	Fish4	101	20	19.80	23	22.77
	Fish5	163	46	28.22	39	23.93
	Fish6	131	26	19.85	25	19.08
	Fish7	73	22	30.14	19	26.03
	Fish8	177	53	29.94	38	21.47
	Fish9	120	40	33.33	24	20.00
	Fish10	187	72	38.50	12	6.42
